# Highly sensitive and selective visual detection of Cr(VI) ions based on etching of silver-coated gold nanorods

**DOI:** 10.1186/s40580-019-0206-1

**Published:** 2019-10-23

**Authors:** Dasom Kim, Eunjin Choi, Chaedong Lee, Yejung Choi, Hoonsub Kim, Taekyung Yu, Yuanzhe Piao

**Affiliations:** 1 Program in Nano Science and Technology, Graduate School of Convergence Science and Technology, Seoul National University, Suwon, 16229 South Korea; 2 Department of Chemical Engineering, College of Engineering, Kyung Hee University, Youngin, 17104 South Korea; 3 Advanced Institutes of Convergence Technology, Suwon, 16229 South Korea

**Keywords:** Gold nanorod, Silver shell coating, Chromium ion sensing, Colorimetric sensor

## Abstract

We report a visual detection of Cr(VI) ions using silver-coated gold nanorods (AuNR@Ag) as sensing probes. Au NRs were prepared by a seed-mediated growth process and AuNR@Ag nanostructures were synthesized by growing Ag nanoshells on Au NRs. Successful coating of Ag nanoshells on the surface of Au NRs was demonstrated with TEM, EDS, and UV–vis spectrometer. By increasing the overall amount of the deposited Ag on Au NRs, the localized surface plasmon resonance (LSPR) band was significantly blue-shifted, which allowed tuning across the visible spectrum. The sensing mechanism relies on the redox reaction between Cr(VI) ions and Ag nanoshells on Au NRs. As the concentration of Cr(VI) ions increased, more significant red-shift of the longitudinal peak and intensity decrease of the transverse peak could be observed using UV–vis spectrometer. Several parameters such as concentration of CTAB, thickness of the Ag nanoshells and pH of the sample were carefully optimized to determine Cr(VI) ions. Under optimized condition, this method showed a low detection limit of 0.4 μM and high selectivity towards Cr(VI) over other metal ions, and the detection range of Cr(VI) was tuned by controlling thickness of the Ag nanoshells. From multiple evaluations in real sample, it is clear that this method is a promising Cr(VI) ion colorimetric sensor with rapid, sensitive, and selective sensing ability.

## Introduction

Cr(III) and Cr(VI) are two main modes of chromium occurrence in aqueous solutions and Cr(III) is one of the essential trace elements in metabolism of glucose and lipids in human body [1]. Cr(III) deficiency may lead to increased cholesterol and blood lipids [2, 3]. Some Cr(III) compounds such as Cr(III) picolinate have been employed as supplemental or alternative medication for diabetes [4]. However, Cr(VI) is well known for its extremely carcinogenic and mutagenic effects and is one of the most critical environmental pollutants [5, 6]. Thus, the U.S. Environmental Protection Agency (EPA) recommends a Cr(VI) concentration of 1 μM (50 ppb) in drinking water as “maximum contaminant level goals” [7]. Cr(VI) has been widely used in many industrial processes including chrome plating, textile industries, leather tanning, and wood preserving. Due to the increasing threat of Cr(VI) exposure in the environment, much more attention has been attracted to highly sensitive and selective assays for the determination of Cr(VI) [8]. A multitude of detection methods for chromium have been reported before and most of these systems include inductively coupled plasma mass spectrometry (ICP-MS) [9], atomic absorption spectrometry [10], inductively coupled plasma atomic emission spectrometry (ICP-AES) [11] and electrochemical methods [12]. Although these methods have high sensitivity and excellent selectivity, they usually require expensive instruments and skilled personnel. Additionally, these methods are not suitable for on-site analysis or in resource-poor settings. Visual methods can be convenient and attractive in many applications because they can be easily observed with the naked eyes without the aid of any advanced instruments [13–15]. Until now, a number of colorimetric sensors have been developed for the detection of ions, small molecules, DNA and protein [16–23]. With advances in nanotechnology, noble metal nanomaterials have been widely studied for various applications such as chemical and biochemical sensing [24–28]. Au and Ag nanoparticles (NPs) are being actively used in visual assays due to their unique optical response which is often called as localized surface plasmon resonance (LSPR) [29–33]. LSPR of Au and Ag NPs are highly dependent on their size, shape, surface charge and local dielectric environment [34, 35]. Taking advantage of these characteristics, lots of colorimetric sensors are based on the aggregation of these metal nanoparticles [36–40]. For example, Chen et al. developed an Au NP-based colorimetric assay for Cr(VI) ions detection using the coordinate covalent bond between meso-2,3-dimercaptosuccinic acid-Au NPs and Cr(VI) [41]. This sensor is accessible for on-site application through translation of the colorimetric signal into the digital signal using a smartphone but still requires procedures for the modification of nanoparticles. Also, this sample cause auto-aggregation and affect the stability of the sensor. Ravindran et al. reported a method for the detection of Cr(VI) using Ag NPs based on the aggregation-induced color change [42]. Although this sensor was easily prepared by unmodified Ag NPs and high selectivity of this sensor has not been thoroughly verified. Recently, Alex et al. developed a non-aggregation based and highly selective Cr(VI) detection method by using etching reactions of Au NRs [43]. Compared with aggregation colorimetric sensors, these non-aggregation-based sensors use simple procedures and show improved linearity. Herein, we designed a novel non-aggregation-based sensor by using silver-coated gold nanorods (AuNR@Ag) as sensing probes for the determination of trace Cr(VI). Our sensing method was based on redox reaction between Cr(VI) ion and Ag nanoshells on the surface of Au NRs. By monitoring the change in the color and the LSPR peak, we could quantitatively determine the Cr(VI) concentration with good linearity. Compared with Au NRs, AuNR@Ag nanostructures produce a much stronger and sharper plasmon resonance, showing considerably superior sensitivity to Au NRs sensor. Under optimized condition, this sensor shows high selectivity towards Cr(VI) over other metal ions and detection limit as low as 0.4 μM. The detection range of Cr(VI) could be tuned by controlling thickness of the Ag nanoshells. Real samples were also tested to confirm the practicability and the results proved that our sensor has good performance in terms of selectivity, sensitivity, linearity, and low limit of detection. This is due to the high extinction coefficient of AuNR@Ag and the signal amplification effect of Ag nanoshells [44–46]. The longitudinal plasmon resonance of AuNR@Ag was blue-shifted as the deposited Ag nanoshells becomes thicker, which could be lead to apparent multicolor change.

## Experimental

### Materials

Gold(III) chloride trihydrate (HAuCl_4_·3H_2_O), cetyltrimethylammonium bromide (CTAB), silver nitrate (AgNO_3_) and l-ascorbic acid (AA) were all obtained from Sigma-Aldrich. Sodium borohydride (NaBH_4_) and sodium hydroxide (NaOH) were purchased from SAMCHUN chemical. CrO_3_, AgNO_3_, Ca(CH_3_COO)_2_, Cd(CH_3_COO)_2_, AlCl_3_, BaCl_2_, CoCl_2_, FeSO_4_, MnCl_2_, NaOH, Pb(CH_3_COO)_2_, LiCH_3_COO, Mg[CH_3_COCHC(O)CH_3_]_2_ were bought from Sigma-Aldrich. Hg(CH_3_COO)_2_, FeSO_4_, ZnCl_2_, KCl, NaOH, Ni(NO_3_)_2_ were procured from SAMCHUN chemical. Ultrapure deionized water was used to prepare all solutions.

### Apparatus

The Au NRs and AuNR@Ag were characterized by high-resolution (HR) transmission electron microscopy (TEM) using a JEOL JEM-2100F instrument, equipped with an energy-dispersive X-ray spectroscopy (EDX) detector. Ultraviolet visible (UV–vis) absorption spectra were collected on a Perkin Elmer Lambda 35 spectrometer. Inductively coupled plasma mass spectroscopic (ICP-MS) measurements were performed with a Perkin Elmer SCIEX NEXION 350D instrument.

### Preparation of gold nanorods (AuNR)

Au NRs were synthesized according to a seed-mediated growth method with slight modifications [47]. In brief, 4.7 mL of 0.1 M CTAB was mixed with 125 μL of 0.01 M HAuCl_4_·3H_2_O under vigorous stirring. Thereafter, ice-cold 30 μL of 0.1 M NaBH_4_ was added to the mixture, and the color of the solution was immediately changed from yellow to brown under vigorous stirring, indicating the formation of the seed solution. The seed solution was continuously stirred for 2 h. To prepare Au NRs growth solution, 4.7 mL of 0.1 M of CTAB, 200 μL of 0.01 M HAuCl_4_·3H_2_O, 30 μL of 0.01 M AgNO_3_ and 32 μL of 0.1 M ascorbic acid were mixed in that order. Finally, 10 μL of seed solution was added the growth solution and leaving this mixture overnight to ensure full growth of Au NRs.

### Synthesis of AuNR@Ag nanostructures

AuNR@Ag nanostructures were prepared using slightly modified procedure that has been described previously [48]. 150, 250 and 350 μL of 0.01 M AgNO_3_ were mixed with 4 mL of Au NRs separately, and then 20 μL of 0.1 M Ascorbic acid and 250 μL of 0.1 M NaOH were sequentially added to increase the pH to 10. Within several minutes, the colors of three solutions were changed, indicating the formation of AuNR@Ag nanostructures. These mixtures were constantly stirred at 30 °C for 2 h.

### AuNR@Ag based sensor for Cr(VI) detection

1 mL of AuNR@Ag was placed in a 4 mL Vial, and a certain amount of 1 M HCl was added to the solution to decrease the pH to 3. Then Cr(VI) containing samples of various concentrations were added to the pH 3 AuNR@Ag colloid solutions. The resulting colloid solutions were stored at room temperature for 10 min. The extinction spectra of the colloid solution were recorded by UV–vis spectrometer. HR-TEM images were observed to characterize the morphology of AuNR@Ag after reacted with Cr(VI) containing samples of various concentrations.

## Results and discussion

### Characterization of AuNR@Ag with different Ag nanoshell thickness

**Fig. 1:**
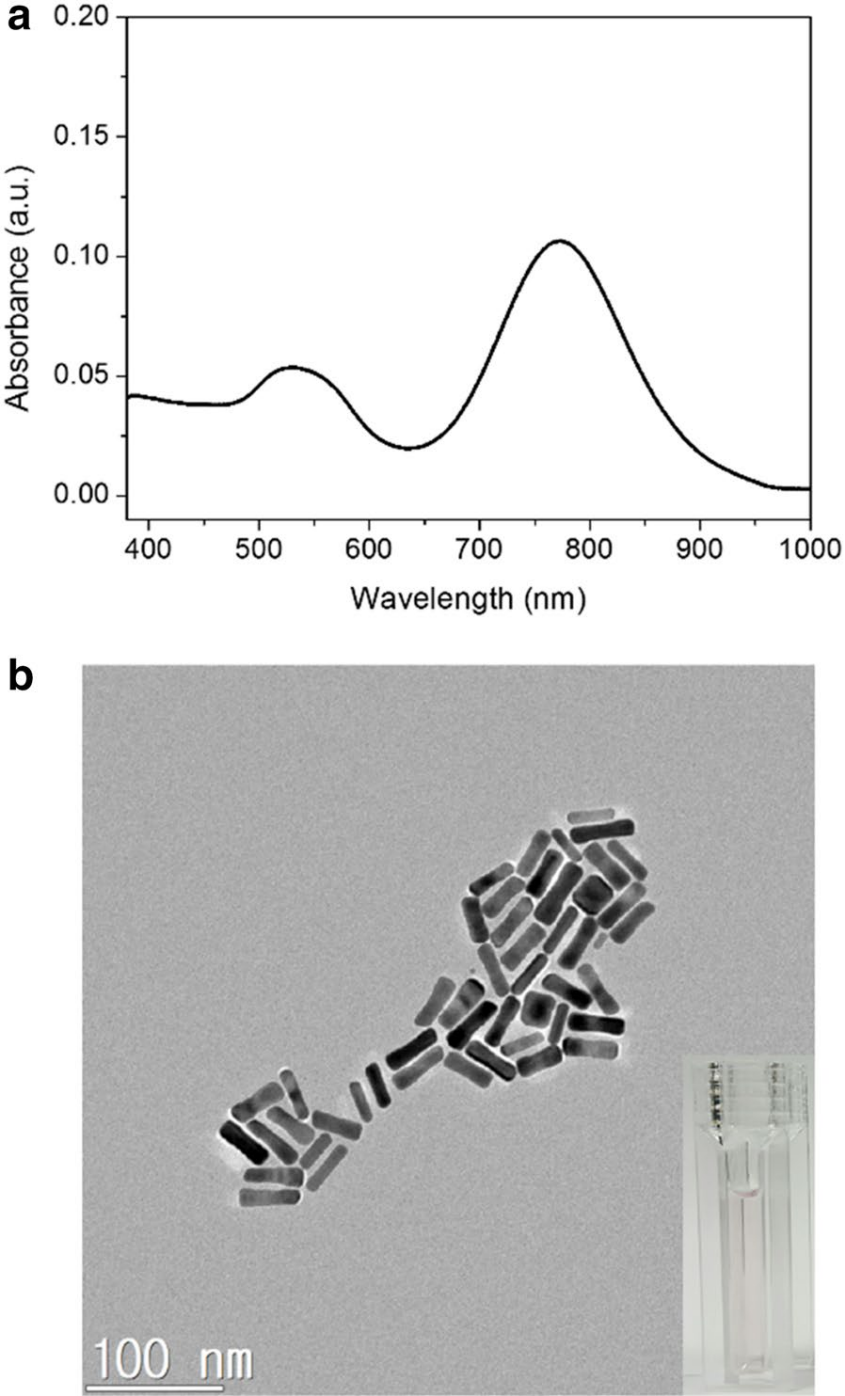
**a** UV–vis absorption spectrum of Au NRs. **b** TEM image of the Au NRs. (Inset: photograph of the Au NR colloid solutions)

**Fig. 2:**
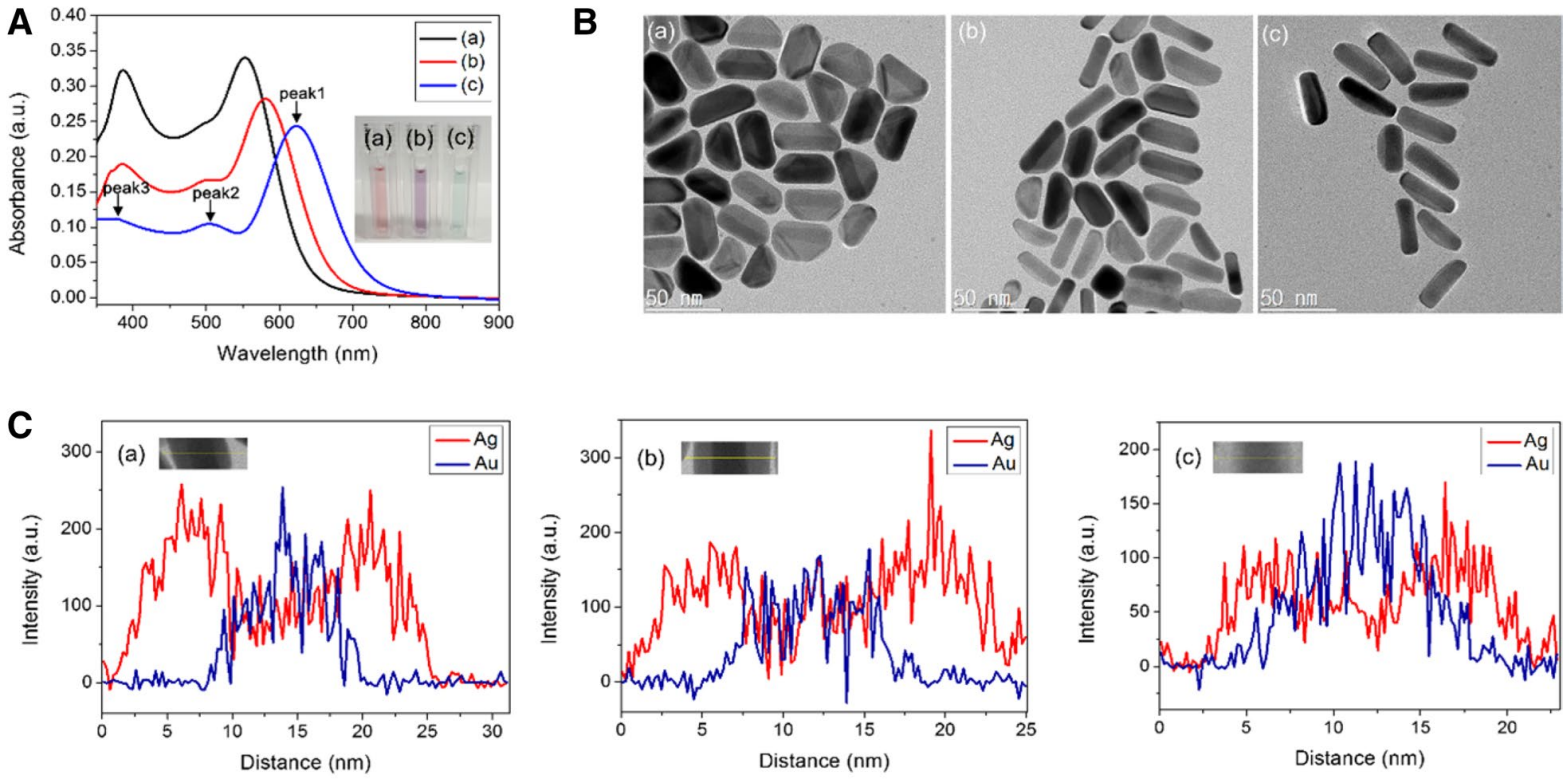
**A** UV–vis absorption spectra (Inset: photographs of the AuNR@Ag colloid solutions with different Ag nanoshell thickness), **B** HR-TEM images and **C** EDX line-scanning profiles along the line in inset **C** of AuNR@Ag with different Ag nanoshell thicknesses obtained by changing the amounts of 0.01 M AgNO_3_ to a 350 μL, b 250 μL, c 150 μL, respectively

UV–vis absorption spectroscopy was used to measure the as-prepared Au NRs. Due to the anisotropic morphology of the Au NRs, the dipole plasmon resonance is split into a transverse plasmon absorption at 532 nm and a longitudinal plasmon absorption at longer wavelength of 774 nm (Fig. 1a). Typical TEM image (Fig. 1b) of the as prepared Au NRs reveal the average aspect ratio to be 3.5 (length and width of the Au NRs were 39.5 ± 3.8 nm and 11.4 ± 1.1 nm, respectively). In order to evaluate the effect of the Ag nanoshell thickness on the sensing performance, Au NRs with Ag nanoshells of different thickness were prepared according to the literature.45 Fig. 2 shows that the thickness of the Ag nanoshell could be tuned by using different amounts of silver nitrate. Compared to the UV–visible spectrum of Au NRs (Fig. 1a), the longitudinal LSPR of the AuNR@Ag (Fig. 2A) exhibited significant blue shifting and enhanced absorbance intensity. As shown in Fig. 2A, with increasing Ag nanoshell thickness, the plasmon resonance peak of the AuNR@Ag was shifted from 590 to 573 nm and 535 nm, and the color of the AuNR@Ag solutions was gradually changed from green to puple and red. Three SPR peaks could be observed and were designed as peaks 1–3 from long to short wavelength. Peak 3 implies that the plasmonic properties of silver were manifested and improved optical properties compared to gold [49]. As Au NRs were coated with silver, peak 1, which was longitudinal SPR peak blue-shifted, was accompanied with enhanced absorbance intensity. Peak 2, related to the transverse SPR peak, remained unchanged and the absorbance intensity of Peak 1–3 was gradually enhanced by increase the thickness of Ag nanoshells [50]. The core–shell nanostructure could be clearly observed in Fig. 2B. As the amount of reduced silver increases, the shape of the particles approached an orange slice-like shape. The HR-TEM observation revealed that the deposited Ag nanoshells on Au NRs in the transverse direction were significantly thicker than in the longitudinal direction. The side facet of Ag nanoshells was varied from 4.6 nm to 6.9 nm and 10 nm by adjusting the amount of silver nitrate (Fig. 2B). Further, the EDS analysis clearly showed that Au was in the core and Ag was in the shell (Fig. 2C). There appeared to be good agreement with HR-TEM observations.

### Sensing mechanism of Cr(VI) ions visual detection using AuNR@Ag nanostructures

**Fig. 3:**
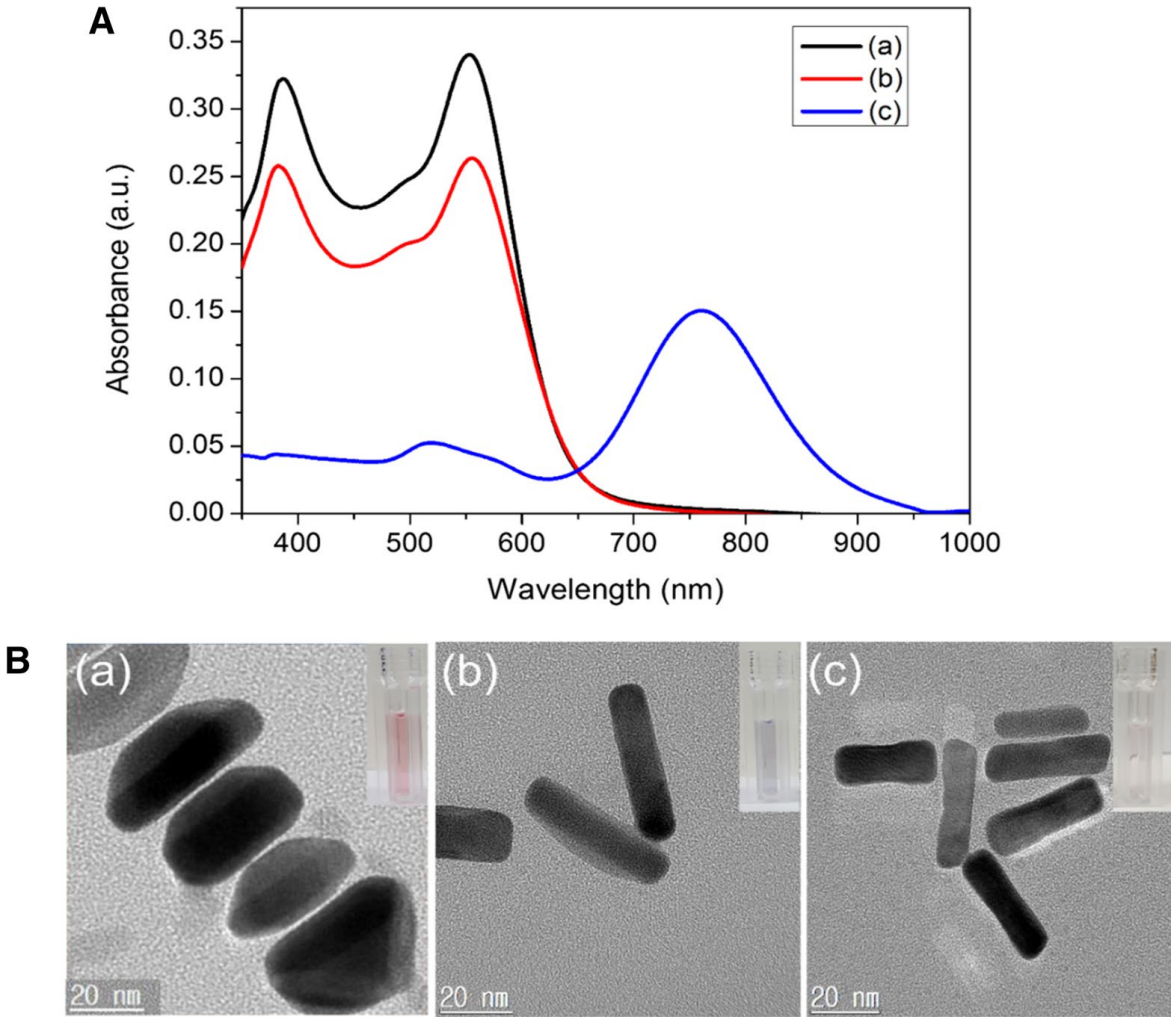
**A** UV–vis absorption spectra and **B** HR-TEM images of: (a) original AuNR@Ag-1 (b) AuNR@Ag after reacted with 10 μM of Cr(VI) and (c) AuNR@Ag after reacted with 60 μM of Cr(VI). (Insets: photographs of each AuNR@Ag colloid solutions)

**Scheme 1:**
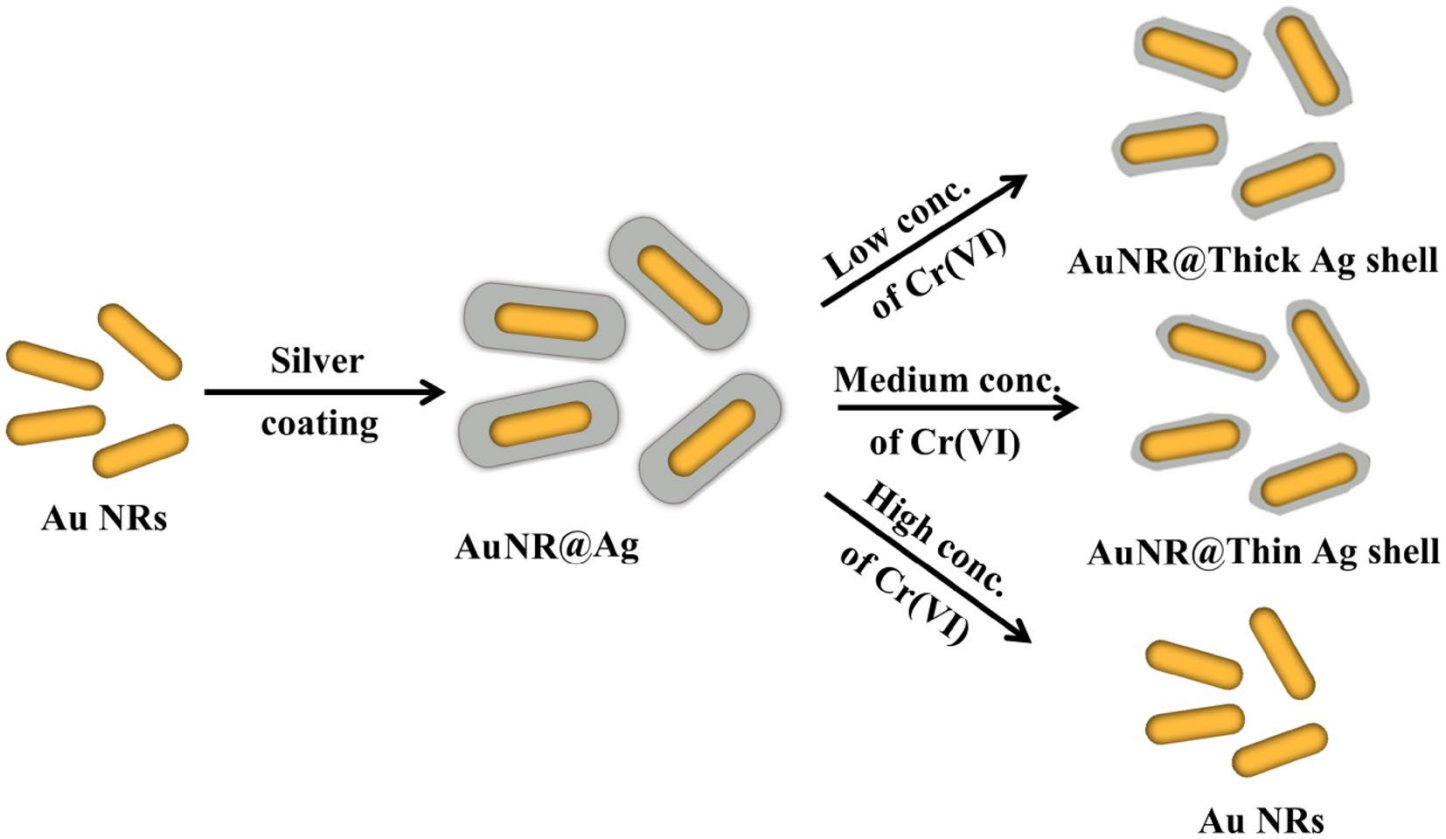
Schematic mechanism of sensing Cr(VI) based on etching of the AuNR@Ag nanostructures

**Fig. 4:**
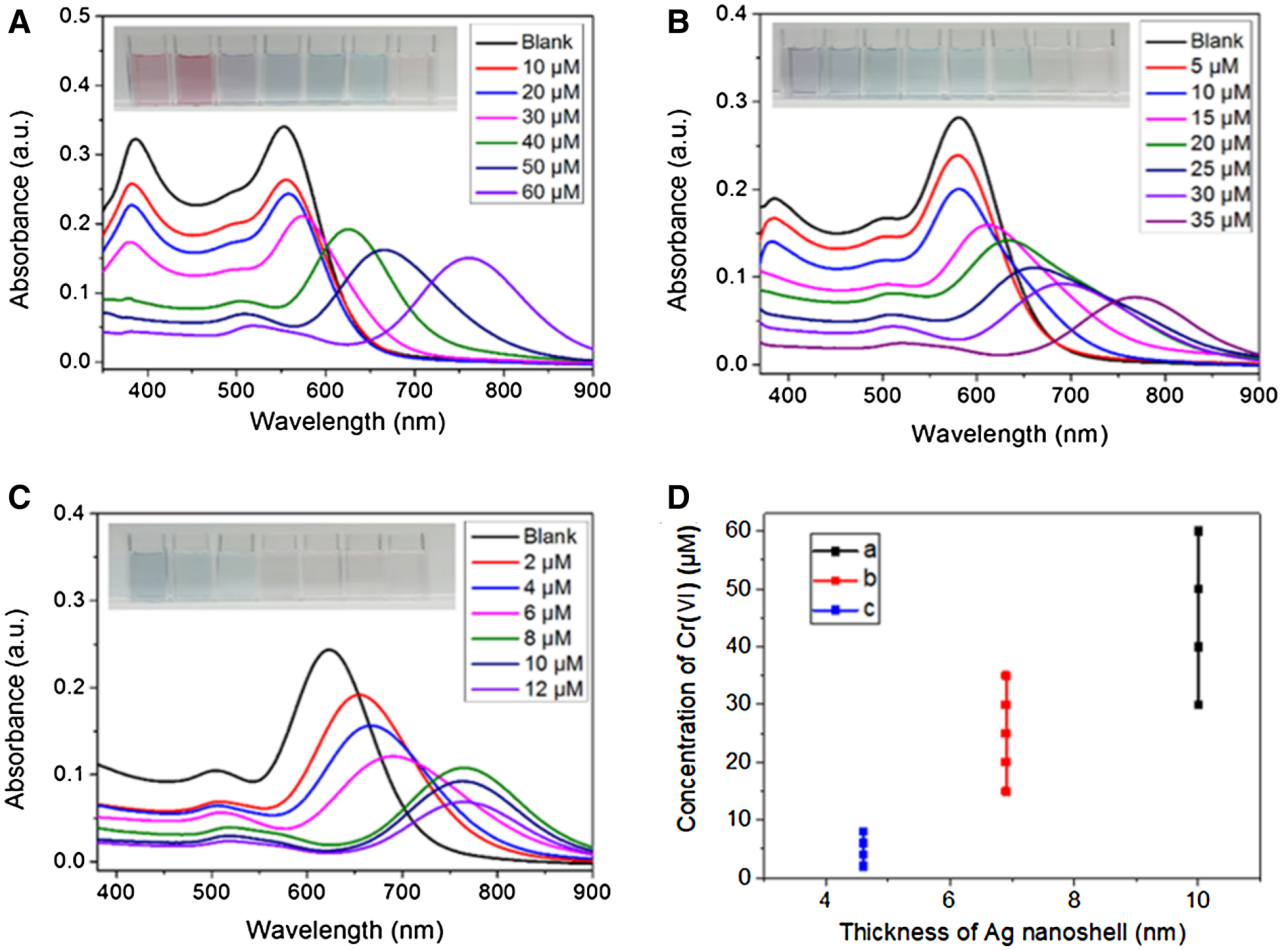
UV–vis absorption spectra of Au NRs coated with Ag nanoshell of different thickness resulting from adding the different amounts of silver nitrate. The absorption spectra of various concentrations of Cr(VI): **a** from 10 μM to 60 μM in AuNR@Ag-1 (Au NRs coated with 350 μL of 0.01 M AgNO_3_), **b** from 5 μM to 35 μM in AuNR@Ag-2 (Au NRs coated with 250 μL of 0.01 M AgNO_3_), **c** from 2 μM to 12 μM in AuNR@Ag-3 (Au NRs coated with 150 μL of 0.01 M AgNO_3_) (Insets: photograph of color change of the AuNR@Ag colloid solutions upon the addition of Cr(VI) with different concentrations). **d** A graph of three distinctive measurement ranges of three nanoparticles with different Ag shell thicknesses (a) AuNR@Ag-1 (b) AuNR@Ag-2 and (c) AuNR@Ag-3. Data of less than 1% change rate were excluded

**Fig. 5:**
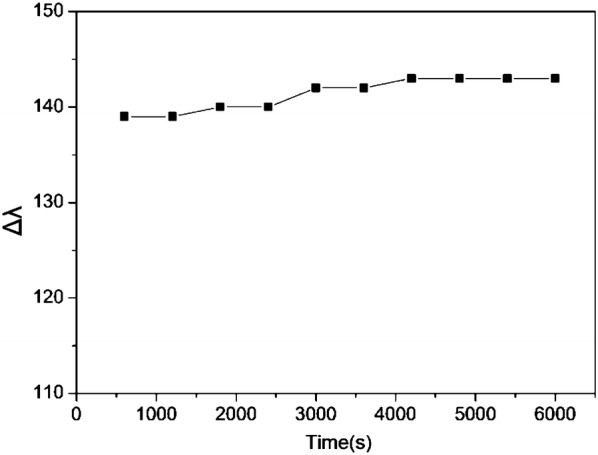
Effect of reaction time on the peak shift of the AuNR@Ag sensing system with adding 10 μM Cr(VI)

In order to demonstrate the sensing mechanism of Cr(VI) ions visual detection using AuNR@Ag, the UV–vis spectra of the AuNR@Ag (obtained by coating 0.01 M AgNO_3_ of 350 μL to Au NRs) as a function of reaction time after mixed with Cr(VI) ions was monitored (Fig. 3A). Furthermore, the HR-TEM images demonstrated that the color change of the AuNR@Ag could be attributed to their morphological transition during the etching process (Fig. 3B). The proposed etching mechanism for the detection of Cr(VI) was illustrated in Scheme 1. A red-shift of the SPR wavelength was due to the decrease of the Ag nanoshell thickness, when the AuNR@Ag was reacted with Cr(VI) in aqueous solution. The etching of the Ag nanoshells could be explained by the theory of standard electrode potential between Ag and Cr(VI). The standard reduction potential of Ag+/Ag(s) and Cr(VI)/Cr(III) are + 0.7993 V and + 1.36 V, respectively [51]. Accordingly, Cr(VI) ions could be reacted with Ag metal spontaneously to decrease the Ag nanoshell thickness of the AuNR@Ag. When the concentration of Cr(VI) was increased, the Ag nanoshell thickness was decreased since more Ag metal was etched away. Accordingly, the significant morphological changes of AuNR@Ag with Cr(VI) proved the viability of using this system to measure trace amounts of Cr(VI). To determine the sensitivity of the sensor, we recorded the UV–vis spectra of the AuNR@Ag in the presence of various concentrations of Cr(VI). Furthermore, we investigate the effect of the Ag nanoshell thickness of the AuNR@Ag on Cr(VI) detection. The AuNR@Ag with different Ag nanoshell thicknesses obtained by changing the amounts of 0.01 M AgNO_3_ to 350 μL, 250 μL and 150 μL, were prepared and called AuNR@Ag-1, AuNR@Ag-2, AuNR@Ag-3, respectively. As the concentration of Cr(VI) increases, the absorbance peak of AuNR@Ag-1 shifted significantly and the color of the AuNR@Ag-1 solution changed from red to purple, green and light violet, sequentially (Fig. 4A). The linear response ranged from 10 to 60 μM and the detection limit was measured to be 2 μM. The absorbance wavelength of AuNR@Ag-2 was slightly shorter than that of AuNR@Ag-1 and the color of the solutions was changed gradually from purple to green and light-violet with the increase of Cr(VI) concentration (Fig. 4B). The Cr(VI) detection system based on AuNR@Ag-2 showed a linear response from 5 to 35 μM, with a detection limit as low as 1 μM. As seen from Fig. 4C, a good linear relationship was also obtained over the range of 2 μM to 12 μM and the limit of detection of Cr(VI) was 0.4 μM. In all experiments mentioned above, with an increase of Cr(VI) ion concentration, a progressive red-shift in the LSPR peak wavelength was observed due to the etching of Ag nanoshells. However, thin Ag nanoshells lead to a quicker red-shift, resulting in improved sensitivity but narrow linear range compared with thick Ag nanoshells. Thick Ag nanoshells expand the linear range but brings down the sensitivity (Fig. 4A). As shown in Fig. 4D, a linear relationship between the absorbance peak and Cr(VI) concentrations in the range of 2 μM to 60 μM was obtained. Among the three samples, we decided to study further about the AuNR@Ag-3, due to its lowest detection limit. The sensing performance of AuNR@Ag-3 was optimized concerning the effect of CTAB and pH. Furthermore, sensing stability was studied over time from 600 to 6000 s (Fig. 5). The results proved that there was no significant change in sensing performance within 10–100 min.

### Optimization of Cr(VI) measurement conditions

**Fig. 6:**
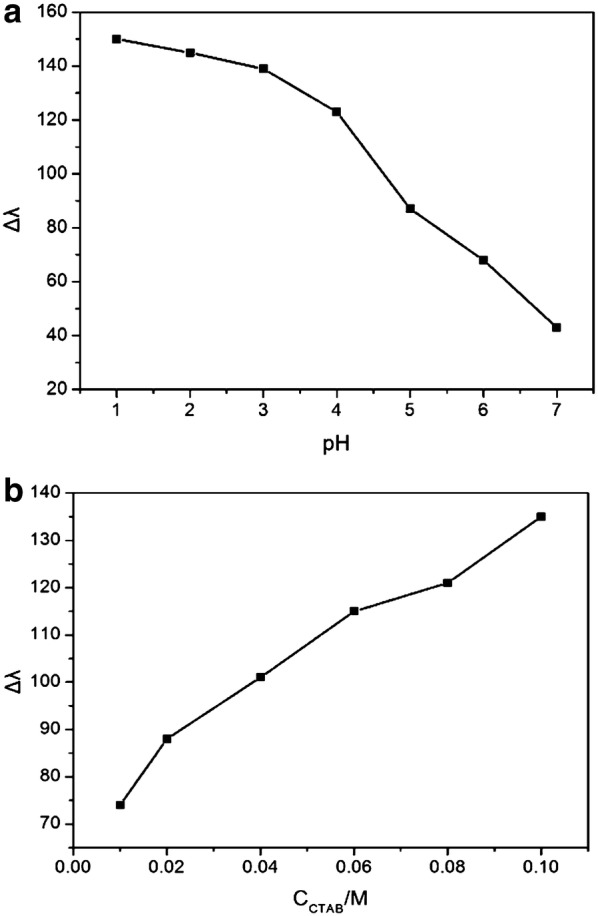
Effect of **a** pH and **b** concentration of CTAB on the peak shift (Δλ) of AuNR@Ag in the presence of 10 μM Cr(VI)

In the pH range of 1–7, the wavelength shift of the AuNR@Ag shows different responses to Cr(VI). As shown in Fig. 6a, the etching rate of the AuNR@Ag increases as the pH value decrease, which was ascribed to the increase of the electron potential and the increase of reduction ability of Cr(VI) as pH decreases. However, at lower pH, the AuNR@Ag become very unstable while at a pH higher than 3, the sensitivity deteriorates. Hence, pH 3 was the ideal working condition for this system. We also studied the effect of the concentration of CTAB, as shown in Fig. 6b. The as-prepared AuNR@Ag was coated with CTAB molecules, which was positively charged. The large amount of CTAB coated on the surface of the AuNR@Ag would be interfere with chemical etching process. It was reported in literature that the positive charges induced by CTAB lowers sensitivity and selectivity of proposed sensing method [52]. Since the CTAB acts as a stabilizer to protect the AuNR@Ag against aggregation, the obtained AuNR@Ag with low concentration of CTAB were unstable [53]. After careful study, the optimum concentration of CTAB was selected as 0.1 M.

### Selectivity of AuNR@Ag based sensor

**Fig. 7:**
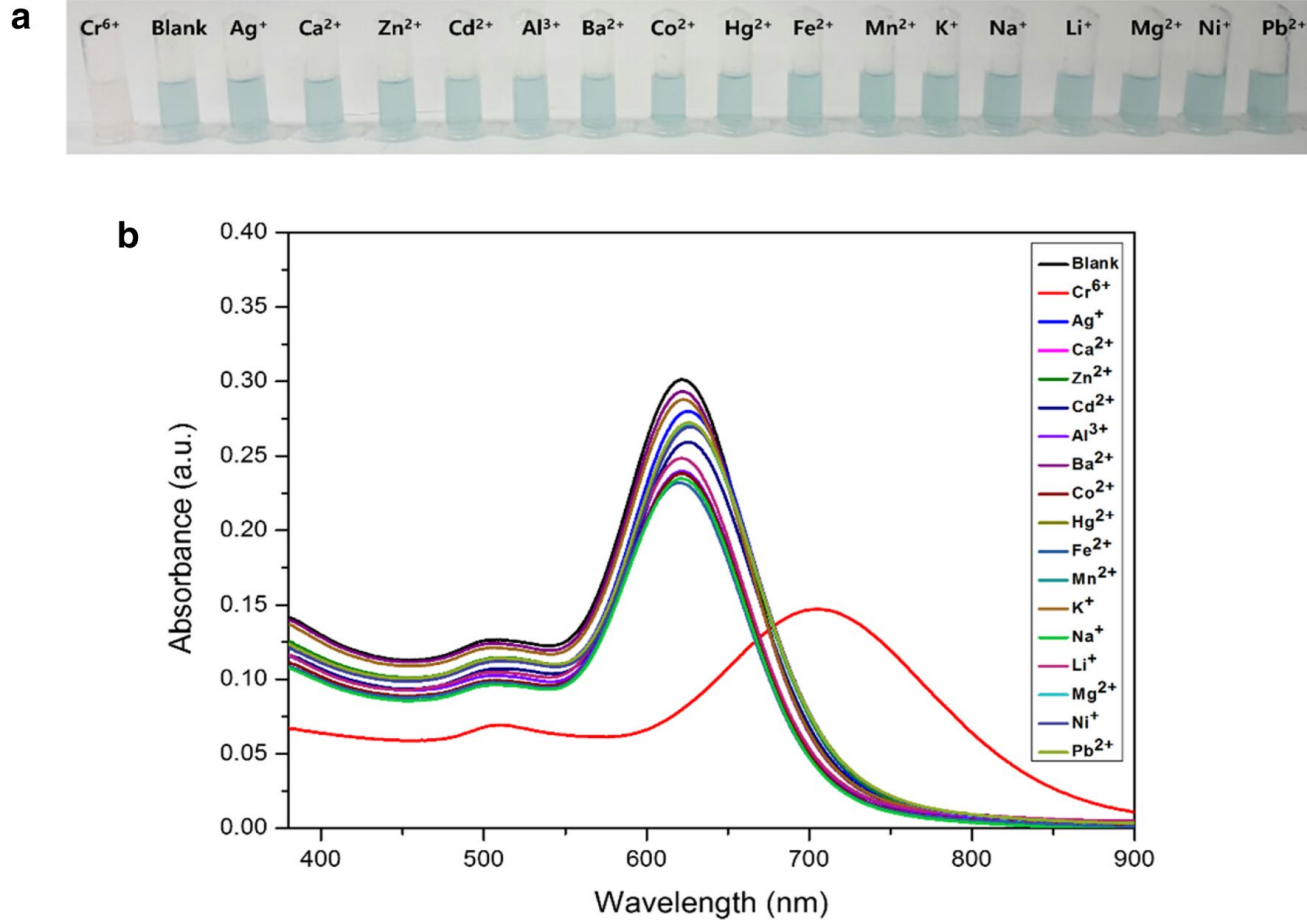
Selectivity of the AuNR@Ag sensing system. **a** Photographic images and **b** the UV–vis spectra of AuNR@Ag based detection systems with various metal ions. (The concentration of Ag^+^, Ca^2+^, Zn^2+^, Cd^2+^, Al^3+^, Ba^2+^, Co^2+^, Hg^2+^, Fe^2+^, Mn^2+^, K^+^, Na^+^, Li^+^, Mg^2+^, Ni^+^, or Pb^2+^ was 110^−4^ M and the concentration of Cr(VI) was 110^−6^ M)

To evaluate the selectivity of the AuNR@Ag based sensor towards Cr(VI) detection, 18 kinds of metal ions (Ag^+^, Al^3+^, Ba^2+^, Ca^2+^, Cd^2+^, Co^+^, Fe^2+^, Hg^2+^, K^+^, Li^+^, Mg^2+^, Mn^2+^, Na^+^, Ni^+^, Pb^2+^, Zn^2+^) were added to AuNR@Ag colloid solutions at the optimized conditions. The 18 kinds of metal ions were tested at a concentration of 100 μM, which was 100 times greater than that of Cr(VI). Figure 7 shows the photographic image and UV–vis spectra of the AuNR@Ag based detection systems with various metal ions. As shown in the photographic image (Fig. 7a), only Cr(VI) ions caused obvious color change from green to light-violet. UV–vis spectrum of the AuNR@Ag based detection system showed a significant wavelength shift and decrease in the plasmonic intensity after the addition of 1 μM Cr(VI).

### Determination of Cr(VI) in real water samples

To evaluate the practical application of our proposed method for Cr(VI) detection, this assay was applied for the determination of trace Cr(VI) in real samples including tap water and lake water (from Gwanggyo, Gyeonggi-do, Korea). Initially, the collected water samples were filtered two times through a membrane to remove any unwanted residue and added with standard Cr(VI) solution. Our proposed colorimetric method was compared with inductively coupled plasma-mass spectrometry (ICP-MS) method. The analytical results were listed in Table 1. The results obtained by both methods were in good agreement, indicating applicability of our designed colorimetric method for the detection of Cr(VI) in real water samples.

**Table 1:** Results for the determination of Cr(VI) ions in different environmental samples

Samples	Added (μg L^−1^)	Found (μg L^−1^)^a^	Recovery (%)	Found by ICP-MS
Tap water	5	5.34 ± 0.1	93.6	5.13
Pond water	5	5.21 ± 0.2	96.0	5.09

^a^Mean standard deviation %

## Conclusions

In summary, a colorimetric method for the determination of trace Cr(VI) ions using AuNR@Ag as sensing probes is proposed. The sensing mechanism relies on the redox reaction between Cr(VI) ions and Ag nanoshells on Au NRs. As the concentration of Cr(VI) ions increased, more significant red-shift of the longitudinal peak and intensity decrease of the transverse peak could be observed. Several parameters such as concentration of CTAB, thickness of the Ag nanoshells and pH of the sample were carefully optimized to determine Cr(VI) ions. Under optimized condition, this method showed a low detection limit of 20 nM and high selectivity towards Cr(VI) over other metal ions, and the detection range of Cr(VI) could be tuned by controlling thickness of the Ag nanoshells. Real sample tests confirm the practicability of the method for environmental field analysis of trace Cr(VI) with good performance.

## Data Availability

The authors have no data to share since all data are shown in the submitted manuscript.

## References

[1] Stearns DM (2000). BioFactors.

[2] Mertz W (1993). J. Nutr..

[3] Glinsmann WH, Mertz W (1966). Metabolism.

[4] Martin J, Wang ZQ, Zhang XH, Wachtel D, Volaufova J, Matthews DE, Cefalu WT (2006). Diabetes Care.

[5] De Flora S, Bagnasco M, Serra D, Zanacchi P (1990). Mutat. Res. Genet. Toxicol..

[6] Zhitkovich A (2005). Chem. Res. Toxicol..

[7] Costa M (2003). Toxicol. Appl. Pharmacol..

[8] Gómez V, Callao MP (2006). TrAC. Trends Anal. Chem..

[9] Gürleyük H, Wallschläger D (2001). J. Anal. At. Spectrom..

[10] Williams CH, David DJ, Iismaa O (1962). J. Agric. Sci..

[11] Liang P, Shi T, Lu H, Jiang Z, Hu B (2003). Spectrochim. ActaPart B At. Spectrosc..

[12] Xing S, Xu H, Shi G, Chen J, Zeng L, Jin L (2009). Electroanalysis.

[13] Wang X, Wei Y, Wang S, Chen L (2015). Colloids Surf. A Physicochem. Eng. Asp..

[14] Jain PK, Huang X, El-Sayed IH, El-Sayed MA (2008). Acc. Chem. Res..

[15] McDonagh C, Burke CS, MacCraith BD (2008). Chem. Rev..

[16] Lee JS, Han MS, Mirkin CA (2007). Angew. Chem. Int. Ed..

[17] Lou T, Chen L, Chen Z, Wang Y, Chen L, Li J, Appl ACS (2011). Mater. Interfaces.

[18] Radhakumary C, Sreenivasan K (2011). Anal. Chem..

[19] Vigderman L, Khanal BP, Zubarev ER (2012). Adv. Mater..

[20] Saha K, Agasti SS, Kim C, Li X, Rotello VM (2012). Chem. Rev..

[21] Feng J, Guo H, Li Y, Wang Y, Chen W, Wang A, Appl ACS (2013). Mater. Interfaces.

[22] Jayabal S, Pandikumar A, Lim HN, Ramaraj R, Sun T, Huang NM (2015). Analyst.

[23] Zhang J, Ouyang J, Ye Y, Li Z, Lin Q, Chen T, Zhang Z, Xiang S, Appl ACS (2018). Mater. Interfaces.

[24] El-Ansary A, Faddah LM (2010). Nanotechnol. Sci. Appl..

[25] Jans H, Huo Q (2012). Chem. Soc. Rev..

[26] Li M, Gou H, Al-Ogaidi I, Wu N, Sustain ACS (2013). Chem. Eng..

[27] Zeng S, Baillargeat D, Ho H-P, Yong K-T (2014). Chem. Soc. Rev..

[28] Li M, Cushing SK, Wu N (2015). Analyst.

[29] Sepúlveda B, Angelomé PC, Lechuga LM, Liz-Marzán LM (2009). Nano Today.

[30] Cao J, Sun T, Grattan KTV (2014). Sens. Actuators B Chem..

[31] Wang X, Chen L, Chen L (2014). Microchim. Acta.

[32] Zhang Z, Chen Z, Qu C, Chen L (2014). Langmuir.

[33] Zhang Z, Chen Z, Pan D, Chen L (2015). Langmuir.

[34] Ray PC (2010). Chem. Rev..

[35] Chen H, Shao L, Li Q, Wang J (2013). Chem. Soc. Rev..

[36] Vilela D, González MC, Escarpa A (2012). Anal. Chim. Acta.

[37] Zhao W, Brook MA, Li Y (2008). ChemBioChem.

[38] Polavarapu L, Pérez-Juste J, Xu Q-H, Liz-Marzán LM (2014). J. Mater. Chem. C.

[39] Niu H, Wang S, Zhou Z, Ma Y, Ma X, Cai Y (2014). Anal. Chem..

[40] Chen L, Li J, Chen L, Appl ACS (2014). Mater. Interfaces.

[41] Chen W, Cao F, Zheng W, Tian Y, Xianyu Y, Xu P, Zhang W, Wang Z, Deng K, Jiang X (2015). Nanscale.

[42] Ravindran A, Elavarasi M, Prathna TC, Raichur AM, Chandrasekaran N, Mukherjee A (2012). Sens. Actuators B Chem..

[43] Alex SA, Satija J, Khan MA, Bhalerao GM, Chakravarty S, Kasilingam B, Sivakumar A, Chandrasekaran N, Mukherjee A (2015). Anal. Methods.

[44] Park K, Vaia RA (2008). Adv. Mater..

[45] Sau TK, Rogach AL, Jäckel F, Klar TA, Feldmann J (2010). Adv. Mater..

[46] Zhang F, Zhu J, Li J-J, Zhao J-W (2015). J. Mater. Chem. C.

[47] Sau TK, Murphy CJ (2004). Langmuir.

[48] Xiang Y, Wu X, Liu D, Li Z, Chu W, Feng L, Zhang K, Zhou W, Xie S (2008). Langmuir.

[49] Jiang R, Chen H, Shao L, Li Q, Wang J (2012). Adv. Mater..

[50] Olson TY, Schwartzberg AM, Orme CA, Talley CE, O’Conneull B, Zhang JZ (2008). J. Phys. Chem. C.

[51] Bratsch SG (1989). J. Phys. Chem. Ref. Data.

[52] Li FM, Liu JM, Wang XX, Lin LP, Cai WL, Lin X, Zeng YN, Li ZM, Lin SQ (2011). Sens. Actuators B Chem..

[53] Zhang Z, Chen Z, Chen L (2015). Langmuir.

